# Identification of priority health conditions for field-based screening in urban slums in Bangalore, India

**DOI:** 10.1186/s12889-018-5194-2

**Published:** 2018-03-02

**Authors:** Sarah Abdi, Avanti Wadugodapitiya, Sandra Bedaf, Carolin Elizabeth George, Gift Norman, Mark Hawley, Luc de Witte

**Affiliations:** 1 Centre for Assistive Technology and Connected Healthcare, School of Health and Related Research, University of Sheffield, The Innovation Centre, 217 Portobello, Sheffield, S1 4DP UK; 2 Zuyd University of Applied Sciences, Nieuw Eyckholt 300, 6419 DJ Heerlen, Netherlands; 3 Bangalore Baptist Hospital, Bellary Road, Hebbal, Bengaluru, Karnataka 560024 India

**Keywords:** Urban slums, Priority health issues, Bangalore, India

## Abstract

**Background:**

Urban slums are characterised by unique challenging living conditions, which increase their inhabitants’ vulnerability to specific health conditions. The identification and prioritization of the key health issues occurring in these settings is essential for the development of programmes that aim to enhance the health of local slum communities effectively. As such, the present study sought to identify and prioritise the key health issues occurring in urban slums, with a focus on the perceptions of health professionals and community workers, in the rapidly growing city of Bangalore, India.

**Methods:**

The study followed a two-phased mixed methods design. During Phase I of the study, a total of 60 health conditions belonging to four major categories: - 1) non-communicable diseases; 2) infectious diseases; 3) maternal and women’s reproductive health; and 4) child health - were identified through a systematic literature review and semi-structured interviews conducted with health professionals and other relevant stakeholders with experience working with urban slum communities in Bangalore. In Phase II, the health issues were prioritised based on four criteria through a consensus workshop conducted in Bangalore.

**Results:**

The top health issues prioritized during the workshop were: diabetes and hypertension (non-communicable diseases category), dengue fever (infectious diseases category), malnutrition and anaemia (child health, and maternal and women’s reproductive health categories). Diarrhoea was also selected as a top priority in children. These health issues were in line with national and international reports that listed them as top causes of mortality and major contributors to the burden of diseases in India.

**Conclusions:**

The results of this study will be used to inform the development of technologies and the design of interventions to improve the health outcomes of local communities. Identification of priority health issues in the slums of other regions of India, and in other low and lower middle-income countries, is recommended.

**Electronic supplementary material:**

The online version of this article (10.1186/s12889-018-5194-2) contains supplementary material, which is available to authorized users.

## Background

While rapid urbanization is emerging as a major challenge globally, the population of urban poor is expected to grow worldwide. Over 800 million people are thought to live in urban slums at present globally, with an estimation to double in the next 30 years [1]. In India, the urban population is expected to grow rapidly from a third to half of its total population by 2030, with a simultaneous expansion of its population of urban poor [2–4].

Within this wider context, Bangalore is a rapidly expanding and developing city that is situated in the southern Indian state of Karnataka, and attracts a large number of migrants from surrounding rural areas [5]. While the Karnataka State Slum Development Board recognizes approximately 600 urban slum areas in Bangalore, informal estimates indicate that there may be up to 1600–2000 slums in the city (*pers comms.*). This includes non-notified slums, which are not recognized formally by the government, and lack many vital services, facilities and amenities [5].

Urban slums are characterised by poverty, housing of poor structural integrity, overcrowding, poor access to water, sanitation and other facilities, and challenging living conditions overall, which impact their inhabitants directly and indirectly [6, 7]. All these factors work in concert to create a unique set of challenges that compromise the health of slum communities. This is illustrated by the fact that urban slum communities often have poorer health outcomes than those in neighbouring urban areas, and even rural areas. The complexity of this situation is exacerbated by the diversity and fluidity of urban slum settings, and given the interplay between the physical and environmental features of slum systems and local socio-cultural contexts, slum communities tend to be particularly vulnerable to a range of health issues, many of which are largely preventable [6–8]. However, slum-based patients have relatively poor access to care, and only tend to come into contact with formal health care services relatively late into their illnesses, if at all [6, 7]. Further, there is a scarcity of information available as to the priority health issues that exist, and are likely to emerge, in these settings, making efforts to prevent, screen for, diagnose and treat the health issues of urban slum communities immensely challenging.

Addressing the health challenges of urban slum communities is becoming an increasingly important consideration in global health [8]. A crucial first step in addressing the needs of these vulnerable communities is to identify, explore, understand and prioritise the major priority health issues they are facing. The present study seeks to identify and prioritise health issues in urban slums in Bangalore, with a wider view of urban slums elsewhere in India, through an exploration of literature and interviews with key stakeholders who work closely with slum communities.

The findings of this study are used to develop a mobile diagnostic and screening toolkit that will help to detect and address the major health challenges in these communities more effectively.

## Methods

**Fig. 1:**
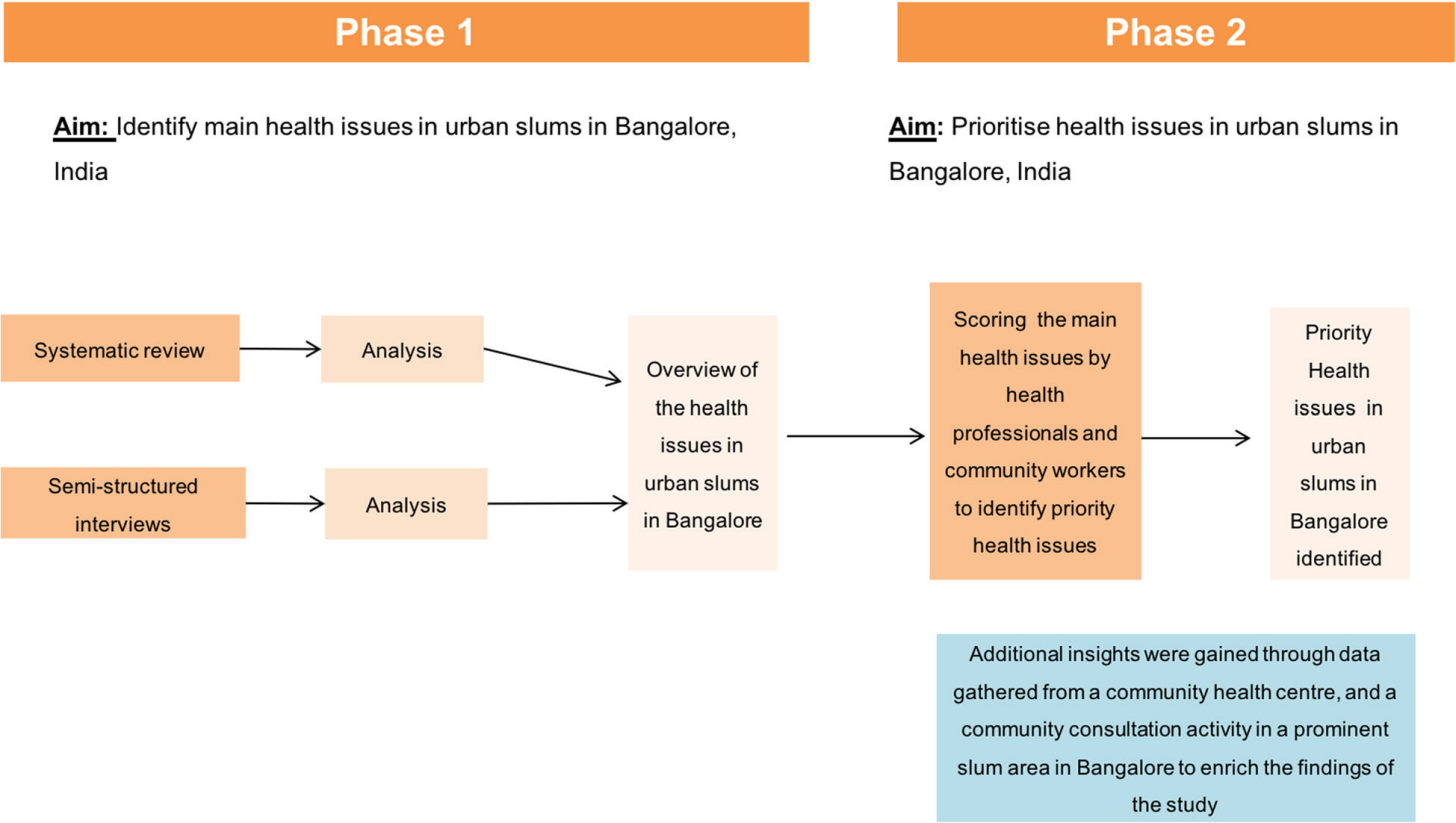
Study design

A two-phased mixed methods design was used in the present study. The aim of Phase I of the study was to identify the health issues reported from urban slums through a systematic literature review and semi-structured interviews with health professionals, community workers and other relevant stakeholders, while Phase II involved the prioritization of these health issues through a consensus workshop. In addition to the data extracted from the literature and interviews, data gathered from a community health centre situated in a slum area in Bangalore and a community consultation activity were used to gain additional insights to complement and enrich the findings of the literature review and interviews. Figure 1 summarises the study design.

### Phase I

#### Systematic literature review

A systematic literature search was performed to identify publications that focus on health issues in urban slums in India. The databases Pubmed, Embase, Cinahl, Cochrane and Google Scholar were searched for records up to August 2016 using the following terms: *health, disease, healthcare* and *health problems* combined in all possible configurations with the terms *slums, urban slums* and *poverty areas*. Two researchers (AW and SA) screened and scored the initial set of titles retrieved based on pre-defined inclusion and exclusion criteria independently. At the beginning of the search, studies from any location were included if they focused on health issues in urban slum setting, so as to get a general overview of the various health issues that exist in the urban slums globally. However, studies were only included in the final analysis if they focused specifically on health issues in urban slum settings in India. Articles that focused exclusively on rural slums, had a veterinary focus, were not written in English, were duplicated, or focussed exclusively on risk factors were excluded. Articles were assigned a score of 0 points if they failed to meet the inclusion criteria, 1 point if they met the criteria partially, and 2 points when they met the inclusion criteria fully. The scores of both researchers were summed and titles scoring a total of two or more points in the first round were examined during the screening of abstracts. When no abstract could be retrieved, the title was scored again. The scores of both researchers were added, and when the total score equalled two or more, the publication was selected for full text analysis. Relevant information, including the health issues mentioned, the specific geographic focus, the target demographic, the study design and the sample size, were extracted from the text. Quality assessment of the publications retrieved was outside the scope of the present study. Cohen’s Kappa was calculated to determine the level of agreement between the two reviewers in the title and abstract assessment**s.**

#### Semi-structured interviews

Semi-structured interviews were conducted to gain insight into the health issues in urban slums with a specific focus on the city of Bangalore. Relevant stakeholders with experience working with urban slum communities in Bangalore, including health professionals and community health workers, were recruited through a snowball sampling strategy. The objectives and procedures of the interviews were described via an e-mail invitation to potential participants. The interviews were conducted via video conference at a time convenient to the participant, and with the aid of a semi-structured interview topic guide. Interviews were conducted on an individual basis, or in small groups, depending on the availability and preferences of participants. The interviews lasted approximately 30–45 min. All participants provided verbal consent prior to the interviews and were given assurances about the maintenance of their anonymity and data confidentiality. All interviews were audio-recorded and summarised following each discussion, with the summaries being shared with the interviewees afterwards for confirmation.

A list of all the health issues mentioned in either the literature or by interviewees was compiled and examined. The health issues were then categorised into four main themes: 1) non-communicable diseases; 2) infectious diseases; 3) maternal and women’s reproductive health; and 4) child health. This categorisation was not mutually exclusive; each health issue could be listed under more than one category if appropriate.

### Phase II

#### Consensus workshop

During the second phase of the study, a workshop was conducted in Bangalore in October 2016 to reach a consensus with the participants on the priority health issues for the local urban slum communities. The majority of participants were interviewees from the first phase, in addition to experts who were invited to participate in the workshop due to their expertise in developing health technologies for low income settings. Preliminary findings from Phase I were shared with the participants. Scoring sheets listing the health conditions identified were prepared for each disease category, and participants were asked to score the health issues in each category based on four criteria: prevalence of the health issue in urban slums (refers to how common it is in urban slum communities they work with); seriousness of the health issue (refers to the extent to which it impacts the overall health of those affected); feasibility of diagnosing/screening for it in the field (refers to the likelihood of being able to screen for the condition in the field); and how beneficial it would be to detect the issue early (refers to whether early screening has the potential to allow better health outcome if these conditions are addressed). These criteria were selected considering the overarching objective of the wider project to which the study belongs (i.e. to develop a screening and diagnostic toolkit to address priority health issues in urban slums). The definitions of the scoring criteria were discussed with the participants prior to the prioritisation exercise so that participants shared a similar perception of each of the criteria. Participants were asked to assign a score between 0 and 5 to each health issue; where 1 is the lowest score, 5 is the highest and 0 is “I don’t know”. For example, assigning a score of 1 to a particular health issue for the seriousness of health issue criterion indicates that the health issue is relatively less serious with reference to the urban slum community. All criteria were given equal weight in the calculation of scores, and the average score assigned to each health issue was calculated by summing the scores assigned to a particular health issue by all participants and dividing this total score by the number of participants. Health issues were then ranked from highest score to lowest in all four categories, based on their average score.

## Results

### Phase I

#### Systematic literature review

**Fig. 2:**
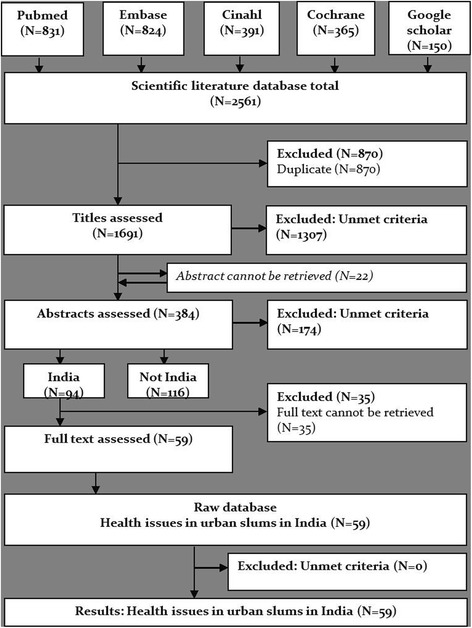
A flow diagram showing the numbers of publications identified and screened for eligibility during the literature review

Figure 2 shows the numbers of publications identified and screened for eligibility during the literature review. The systematic literature search of the databases yielded 2561 references. After excluding duplicates, a list of 1691 titles was created. Cohen’s Kappa for agreement between reviewers SA and AW regarding the title scoring was 0.71, which is substantial [9]. The scoring and selection of titles resulted in a list of 384 abstracts. Cohen’s Kappa for agreement on scoring the abstracts between reviewer SA and AW was 0.80. A total of 94 publications met the inclusion criteria and were eligible for full article assessment. Following the exclusion of studies that did not focus on India and articles for which the full text could not be obtained, a total of 59 articles [10–68] were examined in full.

#### Characteristics of studies

Overall, 59% (*n* = 35) of the studies assessed were observational, of which 27 were cross-sectional. The literature examined included five qualitative studies, five reviews, six studies of mixed methods design, one quasi-experimental study and five studies where the study design did not fit the categories mentioned above. Sample sizes ranged from four to 62,239 and the participants belonged to a range of different age groups. Children were the most studied group in the literature, with 50% of the studies (*n* = 30) reporting health issues exclusively in children. Their health issues were also mentioned in studies that reported health outcomes across a range of age groups (*n* = 9). A total of 12 studies focused solely on women’s health issues, whereas only two studies focused specifically on men’s health problems and one study focused on the older people. With regard to the geographic regions of the studies, 38% of the studies (*n* = 23) were conducted in urban slums located in the Northern region of India, compared to 13 studies in the Western region, nine in the East, three in the South and two in the Central region. A summary of the findings of the literature can be found in an additional file [see Additional file 1].

#### Overview of the health issues identified

Table 1 summarises the health issues identified in the literature and the interviews *(insert after the results of Phase 1*). In total, 57 health issues were identified from the literature (see the first column of Table 1). Diarrhoea was the condition mentioned most frequently in the literature (33 sources), followed by malnutrition (22 sources) and anaemia (11 sources). Several health issues, including diphtheria, leptospirosis and rheumatic heart disease, were each only mentioned in one of the studies examined. Almost half of the health issues (*n* = 28) identified in the literature can be classified as non-communicable diseases, whereas twenty-four issues can be classified as infectious diseases. Several of the health issues identified from the sources examined (*n* = 16) were endocrine, nutritional and metabolic diseases, such as diabetes and nutritional deficiencies. Almost half of the conditions (*n* = 12) mentioned in children were infectious in nature, while a third were nutrition-related (*n* = 8). Diseases of the genito-urinary system, pregnancy-related problems and nutrition-related conditions, such as anaemia and malnutrition, were the main health issues mentioned with regard to women’s maternal and reproductive health.

**Table 1:** Overview of the health issues identified in the literature and the interviews

Health issue^a^	Literature^b^	Interviews^c^
Diarrhoea	33	15
Sexually transmitted infections	4	6
Tuberculosis	4	8
Hepatitis	5	
Typhoid	3	6
Malaria	1	2
Dengue Fever	2	3
Chikungunya	1	8
Tetanus	4	
Sepsis	4	
Measles	3	
Cholera	3	
Parasitic infections	1	3
Meningitis	2	
Leptospirosis	1	
Diphtheria	1	
Infection (unspecified)	1	
Cancer		8
Thalassemia		2
Anaemia	11	13
Malnutrition	22	12
Diabetes	4	15
Thyroid disorders	1	7
Protein and micronutrient deficiencies	1	
Vitamin deficiencies (unspecified)	3	
Vitamin A deficiency	2	
Iodine deficiency	1	
Obesity	9	
Menstrual problems	2	9
Dyslipidaemia	1	
Hypocalcaemia	1	
Rickets	1	
Depression	2	7
Other mental health issues	1	5
Epilepsy	1	
Seizures	1	
Ophthalmological issues	2	1
Dental issues	1	1
Otitis media	1	1
ENT issues	1	
Hypertension	6	16
Coronary heart disease	3	8
Rheumatic heart disease	1	
Renal issues	1	
Pneumonia	4	3
Other respiratory diseases	11	11
Pleural effusion	1	
Other gastrointestinal issues	4	10
Dermatological issues	5	4
Muskuloskeletal issues and arthralgia	2	1
Cervical spondylosis		1
Reproductive tract infections	3	14
Urinary tract infections	1	7
Male reproductive health issues	1	
Obstetric issues	5	3
Low birth weight	5	
Asphyxia	4	
Congenital anomalies and malformations	4	
Fever (unspecified)	4	3
Jaundice	3	2

^a^Other respiratory issues: acute respiratory infections, cough and cold. Ophthalmological issues: eye infections, short sight and diabetic retinopathy. Other gastrointestinal issues: gastroenteritis and vomiting. Obstetric issues: pregnancy induced hypertension, acute partum haemorrhage, severe infections and postpartum haemorrhage. Sexually transmitted infections (STIs): Trichomoniasis, Syphilis, Herpes, HIV and Gonorrhoea. ENT issues: Ear, Nose and Throat infections

^b^the number of times the health issue was reported in the literature

^c^the number of times the health issue was identified as a main health problem in the slums during interviews

#### Semi-structured interviews

##### Description of the participants

A total of 19 participants who work closely with local communities in 5 large urban slums (4 notified, 1 non-notified) in Bangalore were interviewed - six health professionals (provided general health services in community health centres and mobile clinics); ten community health workers (worked in general medical camps and community programmes that focus on women reproductive health and child health); and three other participants (a manager of a health and nutrition initiative and two PhD students who worked on indoor air pollution project and associated health issues).

##### Overview of the health issues identified

A total of 37 health issues were reported as major problems in urban slums in Bangalore during the interviews (see second column of Table 1). It should be noted that not all 37 problems were mentioned in every interview. During the 19 interviews conducted, diarrhoea (mentioned in 17 interviews), anaemia (mentioned in 17 interviews), malnutrition (mentioned in 16 interviews), hypertension (mentioned in 16 interviews), diabetes (mentioned in 15 interviews), and women’s reproductive tract infections (mentioned in 14 interviews) were mentioned most frequently. Three health issues - ophthalmological issues, dental cavities and otitis media - were each mentioned in only one interview. Half of the conditions mentioned during the interviews (*n* = 18) can be classified as non-communicable conditions. One of the participants stated that “chronic conditions like diabetes and hypertension can be found in almost every family”, reflecting the prevalence of such health issues in the context of urban slums in Bangalore. Thirteen of the health issues can be classified as infectious conditions. These conditions were referred to in many interviews as acute conditions, viral diseases, seasonal infections and monsoon related infections. For example, one participant stated, “During our work with people in the slums, we encountered a lot of infectious monsoon-related diseases like chikungunya, malaria and dengue fever”. Children were identified by many participants as one of the groups most vulnerable to health issues in urban slums. Diarrhoea (mentioned in 17 interviews) and malnutrition (mentioned in 12 interviews) were the most frequently reported health problems in children, followed by acute respiratory infections (mentioned in 11 interviews). One of the participants stated that “In child health, malnutrition is a major issue due to lots of reasons like lack of food availability, food insecurity and lack of money to buy food”. Women were also identified as a major group on which the burden of ill health falls, and were reported to be the most frequent visitors to outpatient clinics in the local community health centres. This is illustrated by the observation of by one of the participants that “70 % of (our) patients in the clinic are women”. Reproductive tract infections (mentioned in 14 interviews) and anaemia (mentioned in 11 interviews) were the most commonly mentioned health issues for women.

A total of 60 health issues were identified from the literature and interviews (see Table 1). Diarrhoea, malnutrition and anaemia were the health issues mentioned most frequently in each of the data sources. It should be noted that 27 of the health issues identified from the literature were not mentioned in any of the interviews. Conversely, only three of the conditions mentioned during the interviews - thalassemia, cancer and cervical spondylosis - were not reported in the literature.

### Phase II

A total of fourteen participants - three health professionals, four community workers, four technology experts and three other participants with experience working in health projects in urban slums - completed the prioritization exercise carried out during the consensus workshop in Bangalore. Participants scored a total of 28 health issues in the non-communicable diseases category, 24 health issues in the infectious diseases category, 14 health issues in the maternal and women’s reproductive health category and 28 health issues in the child health category. Table 2 shows the top five priority health issues selected by the participants in each category (*Insert after the results of Phase 2*).

- Non-communicable diseases
  Malnutrition, diabetes and hypertension were selected as the top health priorities in the category of non-communicable diseases in terms of both their prevalence and the feasibility of screening for them in the field. However, malnutrition was not seen as one of the top five health issues in terms of the potential benefits of screening for it early in the field, while neither hypertension nor diabetes was selected as a top priority with regard to seriousness of the health condition. Some conditions scored highly on one or two criteria, but not on other criteria. Cancer, renal problems and coronary heart diseases, for instance, were amongst the top five priority health issues in terms of their seriousness, but not with regard to any other criteria.
- Infectious diseases
  Dengue fever was identified as one of the top five infectious health issues for all four criteria, followed by diarrhoea and sexually transmitted infections (STIs), which were prioritised in terms of three criteria. The remaining health issues in this category were selected as top health priorities in one or two criteria only.
- Maternal and women’s reproductive health
  Malnutrition and anaemia were scored as the top two priority health issues in the maternal and women’s reproductive health category, in terms of all four criteria. Vitamin deficiencies was perceived as a highly prevalent and serious, but was not selected as one of the top priorities in terms of feasibility of screening and benefits of early screening in the field. Other conditions were prioritised under only one criterion, including obesity for the prevalence of the health issue criterion, and urinary tract infections (UTIs) for the feasibility to screen for in the field criterion.
- Child health
  Diarrhoea was selected as the top health priority for children on all criteria, followed by malnutrition, anaemia and sepsis.

**Table 2:** Top five health issues prioritised in each disease category

Ranking	Prevalence of the health issue	Seriousness of the health issue	Feasibility of screening and/or diagnosis in the field	Potential benefits of early detection
a) Non-communicable diseases				
1	Malnutrition	Malnutrition	Hypertension	Otitis media
2	Diabetes	Cancer	Diabetes	Hypertension
3	Dental cavities	Renal issues	Malnutrition^a^	Ophthalmological issues
4	Hypertension	Other respiratory issues^a^	Ophthalmological issues^a^	Diabetes^a^
5	Other respiratory issues	Coronary heart diseases^a^	Dental cavities	Jaundice^a^
b) Infectious diseases				
1	Diarrhoea	Diarrhoea	Dengue fever	Tuberculosis
2	Dengue fever^a^	Tuberculosis	Other respiratory issues	Dengue fever
3	Dermatological issues^a^	Dengue fever^a^	Dermatological issues^a^	Diarrhoea
4	Other gastrointestinal issues	Renal issues^a^	Renal issues^a^	Sexually transmitted infections^a^
5	Sexually transmitted infections	Sexually transmitted infections	Other gastrointestinal issues	Other respiratory issues^a^
c) Maternal and women’s reproductive health				
1	Anaemia	Malnutrition	Anaemia	Anaemia
2	Malnutrition	Anaemia	Malnutrition	Malnutrition
3	Vitamin deficiencies (unspecified)^a^	Sexually transmitted infections	Obstetric issues	Sexually transmitted infections^a^
4	Obesity^a^	Vitamin deficiencies (unspecified)^a^	Dermatological issues	Tuberculosis^a^
5	Menstrual problems^a^	Obstetric issues^a^	Urinary tract infections	Menstrual problems
d) Child’s health				
1	Diarrhoea	Diarrhoea	Diarrhoea	Diarrhoea
2	Malnutrition	Malnutrition	Tetanus	Malnutrition
3	Anaemia	Anaemia	Sepsis	Anaemia
4	Protein and micronutrient deficiencies	Sepsis	Measles	Sepsis
5	Vitamin deficiencies (unspecified)	Congenital anomalies and malformations	Cholera	Cholera

^a^equal scores

#### Additional insights

Aside from the examination of literature and stakeholder interviews, additional insights were gained through data gathered from a community health centre, and a community consultation activity in a prominent slum area in Bangalore, in order to enrich the findings of the study.

The data from the community health centre consisted of the numbers of patients that visited the clinic, as well as an indication of the nature of their ailment, and indicated that the majority of the 23,056 patients examined at the clinic over a period of two years were reported to have complaints related to diabetes, hypertension and/or heart disease (61.4%), while 36.2% were reported to have central nervous system-related complaints, 26.9% musculoskeletal issues, 26.3% dental issues, 11.9% gynaecological issues, 11.2% ophthalmological issues and 10.8% respiratory issues. Other health issues reported from the clinic data include gastrointestinal issues, dermatological issues, obstetric issues, issues requiring surgery, endocrine issues, urological issues, nutritional issues and psychiatric issues.

The community consultation session sought to explore the local community perceptions of health and the health issues they face. Approximately 30–40 people participated in this activity, during which they were invited to share their thoughts on the main health issues faced by them, their families and neighbours. Overall, the perspective communicated by the local community initially was that they do not face many health issues. However, following further discussion, the health complaints that were reported during the community consultation session include cold, fever, dental caries and infections, asthma, otitis media, respiratory tract infections, diabetes, blood pressure, anaemia, spontaneous abortion/miscarriages, epilepsy, tuberculosis, kidney stones, thyroid problems, nephrotic issues, accidents, stroke and reproductive morbidities. Although diarrhoea was mentioned, the community did not see this as a major problem below one year of age. Further, they indicated that they only go to the hospital when there is a major crisis. It should be noted that these conditions were referred to using local terminology, with the discussion facilitated and interpreted by doctors and health workers who work with the community on a regular basis.

## Discussion

This study aimed to identify the priority health issues in urban slums in Bangalore, India. Malnutrition and anaemia were identified as top priorities for women and children. This finding was expected, as these conditions top the list for factors contributing to the disease burden in India overall [69], particularly in children and women living in areas of poverty [70, 71]. Diarrhoea was also selected in the child health and infectious disease categories. This finding was also expected, as diarrhoeal diseases are one of the top five leading causes of death in children and in the population overall [69], in India, with slum dwellers being disproportionately susceptible to these conditions due to the challenging living conditions they endure, including extreme poverty and poor sanitation. It should be noted that diarrhoea, malnutrition and diabetes were also the most frequently mentioned conditions encountered in the interviews and the literature examined during this study.

Nevertheless, the frequency of being reported in the literature is not necessarily a reflection of the prevalence of a particular health issue among the urban slum communities in Bangalore. On the contrary, it might indicate a publication bias or the prominence of that health issue in a region or at a given point in time. In this study, several conditions were identified as priorities in the workshop despite not receiving much attention in either or both of the data sources during the first phase of the study. For instance, dengue fever was selected as a top priority in the infectious diseases category for all four criteria during the prioritisation exercise. Given that it is a seasonal or epidemic health issue, it is possible that seasonality was taken into account when scoring the criteria for this condition. Sepsis was also selected as a priority health condition in the child health category for three criteria, which is in line with the WHO statistical profile of India that lists neonatal sepsis as one of the main causes of mortality in children under the age of five [69]. Further, although diabetes and hypertension were not mentioned in a large number of the publications examined, they were selected as top priorities in the non-communicable disease category, reported for the majority of patients visiting the community health centre, and listed among the main health complaints during the community consultation session. Indeed, non-communicable diseases, such as diabetes and hypertension are emerging as major public health problems in several developing countries including India and are recognised to exist in slum areas.

This study also demonstrated an equal distribution of health issues across infectious and non-communicable disease categories in both data sources examined in the first phase of the study, which might indicate that both types of health issues are of almost equal importance in urban slums in Bangalore. This is contrary to the common belief that infectious diseases would be more dominant in these areas [72] due to poor living conditions like lack of clean water, and indicates that the double burden of disease that characterises the health status of people in many low and lower middle income countries is also reflected within urban slum communities.

Women and children were found to be the most studied demographic groups in the literature review, and were also identified by interviewees as the most vulnerable groups in the slums. This is consistent with several national and international reports, which reveal large inequities in the health and mortality rates of women and children in Low and Lower Middle-Income Countries (LMICs).

It would have been beneficial to compare the results of this study with priority health issues in slums in other parts of India, and the world, to understand the similarities and variations of health issues in different settings. However, studies on priority health issues in urban slums are scarce. The vast majority of studies that examine the health status of urban slum dwellers focus on reporting the prevalence of specific health issues or causes of ill health in slums and so do not provide a suitable comparison.

One of the main strengths of this study is that it followed a mixed methods approach, which helped to gain a better understanding of the health issues in urban slums in Bangalore than would be obtained by a single method of data collection. For instance, several health issues identified from the literature were not mentioned in any of the interviews, and would have been missed in the prioritisation activity if only one data source was used. Further, the perceptions of the local slum community expressed during the community consultation session with regard to their health provided additional, and unexpected insights, that would have otherwise been missed. For example, they did not regard diarrhoea as a serious health problem among children below the age of one year, despite the fact that diarrhoea can be life threatening at this age and was identified as a priority health issue for the local urban slum community during the prioritization exercise. Certain health issues can become “normalised” due to their high prevalence and close association with the challenging living conditions in slums, and the fact that there is often little that slum dwellers can do to address them. Therefore, it was important to engage with local communities, and better understand their perceptions of health and priorities in order to develop future interventions based on their needs.

One of the limitations of the study was the sampling strategy utilized for the interviews. Snowball sampling was used to recruit participants as it would have otherwise been difficult to identify interviewees within the local context. This might have introduced some bias into the health issues identified in the interviews as the sample is not necessarily a clear representation of all people working in health-related projects in the slums. However, it is anticipated that using other data sources (e.g. examination of the literature) to identify the health issues in urban slums may have overcome this bias partially. Also, the professional background of the participants might have influenced the health conditions identified, particularly in the case of the technology experts as their area of expertise was different to the remaining participants. However, by comparing their results to the rest of the group, no considerable difference was observed which may be due the fact that many of them were familiar with urban slum settings and health challenges of these settings through other initiatives.

Although there are similarities in the living conditions of urban slums in different regions, and the findings of the present study may be useful to stakeholders working with urban slum communities outside of Bangalore, extrapolation to other slums within India and elsewhere should be done with caution, as all slums have their own unique characteristics, challenges and health issues. Also, the priority health conditions identified were based on criteria relevant to the objective of the wider project to which this study belongs (i.e. to develop a screening and diagnostic toolkit to address priority health conditions in urban slums). This might need to be taken into consideration when interpreting the results of the prioritisation exercise as there might be other criteria that are relevant to the local communities.

The study was of exploratory in nature and aimed to identify and prioritise health conditions occurring in the urban slums of Bangalore. Hence, identifying and addressing factors contributing to the burden of ill health in these communities was beyond the scope of this study. However, it is noteworthy that this study is linked to a large initiative called “Health in Slums” which aims at improving the health and wellbeing of urban slums communities in Bangalore and include other projects that address some of the challenging living conditions faced by these communities.

Following the completion of the present study, a preliminary prototype of the toolkit was developed by the project team. This initial prototype has been field tested and evaluated through a pilot study in one large slum, and it is expected that it will be refined and developed further through future projects.

## Conclusions

Urban slums have unique characteristics in terms of the challenging living conditions that make their inhabitants vulnerable to specific health issues. Identification of the priority health issues in urban slums is a necessary step in ensuring that any intervention that aims to improve the health status of the local urban slum communities is based on their needs, and that resources are allocated to the most important health issues. In this study, several health issues were selected as top priorities for the urban slums of Bangalore, India. The results of this study can be used to inform the design of interventions to improve the health status of local urban slum communities in Bangalore. Further, its findings are being used by the research team to develop a screening and diagnostic toolkit to address the priority health issues identified through the present study.

## Additional file

Additional file 1: Summary of the findings of the literature. 59 articles were examined in full. The following table summarises the findings of these articles which include the objective of the study, its location, the target demographics, the study design, the sample size and the health issues mentioned in each study. (DOCX 29 kb)

## Abbreviations

LMICs: Low Middle Income Countries
STI: Sexually Transmitted Infections
UTI: Urinary Tract Infections
WHO: World Health Organisation
