# Supplementary material for: Identification of priority health conditions for field-based screening in urban slums in Bangalore, India
Source: BMC Public Health. 2018 Mar 2;18:309. doi: 10.1186/s12889-018-5194-2 (PMC5833095; doi:10.1186/s12889-018-5194-2)
Supplement: Supplementary file 1 — Summary of the findings of the literature. 59 articles were examined in full. The following table summarises the findings of these articles which include the objective of the study, its location, the target demographics, the study design, the sample size and the health issues mentioned in each study. (DOCX 29 kb) [file 12889_2018_5194_MOESM1_ESM.docx]

**Additional file 1:** Summary of the findings of the literature

| **No.** | **Source** | **Location** | **Objective (s)** | **Target demographic** | **Study design** | **Sample size** | **Health issue(s) mentioned** |
| --- | --- | --- | --- | --- | --- | --- | --- |
| 10 | Acharyya *et al.* (2014) | North 24 Parganas District, West Bengal | To estimate the prevalence of non-communicable disease risk factors in the slums in North 24 Parganas. | Adults | Cross sectional | 1052 | Hypertension Obesity |
| 11 | Agarwal *et al.* (2007) | Balmiki Basti, Dehli | To evaluate the socio-demographic correlates and barriers of maternal health-care utilization amongst married women aged 15-45 years living in a slum in Delhi. | Pregnant women at the time of interview or had delivered within the last 1 year Aged 15-45 years old | Qualitative study | 100 | Anaemia  Hypertension Tuberculosis |
| 12 | Agarwal and Taneja (2005) | Indore, Madhya Pradesh | To describe the rationale for identifying the most vulnerable among the urban poor, while planning city level child health interventions. | Children | Review article | - | Diarrhoea Malnutrition |
| 13 | Awasthi and Pande (1998) | Lucknow | To assess sexual behavior patterns and knowledge of STD among underprivileged 15 to 21 old boys. | Adolescents (Boys) | Cross sectional | 221 | Sexual transmitted infections |
| 14 | Awasthi *et al.* (1996) | Lucknow | To provide an overview of the causes of death in the preceding 3 years in a slum population of children under 5 years old. | Children (0-5 years old) | - | 24,196 | Anaemia Diarrhoea Fever Malnutrition Measles Pneumonia Tetanus |
| 15 | Banerjee *et al.* (2012) | An urban field practice area situated at Bhosari, near Landevadi slum | To study access to health services among slum dwellers and rural population. | Mixed (all age groups) | Mixed design | 865 | Chikungunya Coronary heart diseases Dermatological issues Diabetes Diarrhoea Diphtheria Fever Hypertension Jaundice Malaria Malnutrition Musculoskeletal and arthritis Obesity Other respiratory issues Tuberculosis Typhoid |
| 16 | Bapat *et al.* (2012) | Mumbai | To classify neonatal deaths according to international cause-specific criteria. | Children (newborn) | Qualitative study | 11,305 | Anaemia Congenital anomalies and malformations Infection Obstetric issues Tetanus |
| 17 | Basu and Basu (2000) | Pushta jhuggi-jhompri cluster in New Seelampur, East Delhi | To assess the impact of the environment (physical, social, economic) of urban slum on the health and well-being of slum dwellers, particularly women. | Women | Mixed design | 150 households | Anaemia Dermatological issues Gastroenteritis Musculoskeletal and arthritis Other respiratory issues Tuberculosis |
| 18 | Bhandari *et al.* (2002) | Two urban slums in Delhi | To obtain insights into the processes underlying infant deaths. | Children (newborn) | Cross sectional | 4000 | Asphyxia Congenital anomalies and malformations Diarrhoea Meningitis  Pneumonia |
| 19 | Bhanderi and Kannan (2010) | The city of Rajkot in Gujarat state | To assess the reproductive health of ever married women aged 15–49. | Women | Mixed design | 1,046 eligible women from 826 households were interviewed | Obstetric issues Sexually transmitted infections |
| 20 | Bhargava *et al*. (1991) | Three urban slums in Delhi, Calcuta, and Medras | To identify the families at greater risk from health and nutritional point of view; to study high risk urban slum and rural community ever married women of reproductive age period from pre-prganant state to conception, and termination of pregnancy and relate to fetal outcome to social, enviromental, maternal nutritional and biologocal factors; and to obtain information on utilisation of available maternal and child health services during antenatal period of pregnancy, delivery, child birth, post partum period and for infant care. | Women (13-49 years old) | Cross sectional | 17754 urban households which included 18006 ever married women | Anaemia Asphyxia Low birth weight Obstetric issues Other respiratory infections Sepsis Tetanus Uniary tract infections |
| 21 | Bhilwar *et al.* (2015) | An urban resettlement colony of Gokulpuri in the North-East district of Delhi | To document the prevalence and determinants of Reproductive Tract Infections in married women (15-49 years) residing in an urban slum in Delhi, India. | Women (15-49 years old) | Qualitative study using semi-structured questionnaires | 802 | Menstrual problems Reproductive tract infections |
| 22 | Chadha *et al.* (2014) | Urban slums, non-slum urban areas and rural areas across two districts of Delhi | To compare the prevalence of different forms of otitis media in the three main types of settlements, i.e. urban slums, non-slum urban and rural areas, which are inhabited by people living in Delhi. | Children (0-15 years old) | Cross sectional | 3000 | Otitis Media |
| 23 | Chauhan *et al.* (2011) | Urban slums of Ahmedabad | To assess the prevalence of Vitamin A deficiency among school children of 5-15 years in urban slums of Ahmedabad, India. | Children (5-15 years old) | Cross sectional | 1000 | Vitamin A deficiency |
| 24 | Das *et al.* (2012) | Mumbai | To describe the proportions of underweight, stunting, and wasting in young children in urban slums; to test the hypothesis that underweight (low weight for age) is largely explained by low height for age; and to examine the relationships between z scores and age. | Children | Other (two linked datasets for the analysis: a large primary dataset collected through prospective registration of births in 48 slum areas (each of at least 1000 households), and follow-up of a subsample of children) | 1941 | Low birth weight Malnutrition |
| 25 | Dhar (2014) | Not specified | To discuss Coronary Heart Disease Risk of Slum Dwelling Residents in India. | Adults | Review article | - | Coronary Heart Diseases |
| 26 | Diamond-Smith *et al.* (2016) | Chandigarh City | To ascertain the determinants of persistence maternal anemia in Chandigarh. | Pregnant women (18-35 years old) | Mixed design | Three focus group discussions with pregnant women from different socioeconomic group. Interviews of 120 pregnant women | Anaemia |
| 27 | Emmel and D'Souza (1999) | Mumbai | To discuss the health effects of forced evictions in the slums of Mumbai. | Children (15-29 months old) Prepubescent children | Article (no clear design) | 70 of children aged 15-29 months old  100 prepubescent children | Malnutrition Protein deficiency  Rickets Vitamin A deficiency |
| 28 | Fernandez *et al*. (2003) | Not specified | To discuss urban slum-specific issues in neonatal survival. | Children (Newborn) | Review article | - | Anaemia Asphyxia Low birth weight Malnutrition Sepsis Tetanus |
| 29 | Garg *et al.* (2001) | A slum - Balmiki Basti, located in the vicinity of Maulana Azad Medical College | To estimate the prevalence of Reproductive Tract Infections (RTIs) by symptom complexes in a slum population of Delhi; to study the risk factors associated with RTI; and to study the health care seeking behaviour of urban population in relation to RTI. | Women (15-45 years old) | Qualitative study using semi-structured interviews | 231 | Reproductive tract infections |
| 30 | Garg *et al.* (2007) | Bawana, Sanjay Amanr Colony, Tilak Nagar Colony & Lal Bagh | To study the prevalence of STIs among males; and to assess the level of awareness of STIs amongst males of the reporductive age group residing in an urban slum of Delhi. | Males (15-49 years old) | Cross sectional | 196 | Male reproductive problems Sexually transmitted infections |
| 31 | Gaur *et al*. (2013) | Covered sub-samples from eight mega-cities of India, namely Mumbai, Delhi, Kolkata, Chennai, Hyderabad, Indore, Meerut and Nagpur | To answer the following question: whether duality in women’s nutritional status cuts across a mega-city or whether policy should treat slum areas differently from non-slum areas. | Women (15-49 years) | Cross sectional | 19,448 | Malnutrition Obesity |
| 32 | Ghosh and Shah (2004) | Not specified | To discuss nutrition problems in urban children in India. | Children | Review article | - | Anaemia Iodine Deficiency Disorder  Malnutrition Vitamin D Deficiency |
| 33 | Ghosh-Jerath *et al.* (2015) | Three urban slums in India’s national capital, Delhi (namely CPJ, Buland Masjid and Chanderpuri were from three constituencies of North-East district of Delhi) | To explore Antenatal care utilization, dietary practices and nutritional outcomes in pregnant women and recently delivered women in urban slums of North-east district of Delhi, India. | Women | Cross sectional | 6092 households participated in the HH survey. 184 pregnant women and 160 mothers participated in the detailed survey. | Anaemia Vitamin D deficiency |
| 34 | Gill *et al*. (2014) | Urban slum areas of Amritsar city in Punjab | To assess the prevalence of malnutrition among infants; and to determine sociodemographic correlates impacting the nutritional status of infants in urban slum areas of Amritsar city. | Children | Cross sectional | 210 | Malnutrition Obesity |
| 35 | Gomber *et al.* (2003) | Urban slums located near the Guru Teg Bahadur (GTB) Hospital, Delhi | To determine the prevalence of anaemia among school children aged 5 to 10.9 yr from urban slums; and to define the etiology of nutritional anaemia among this vulnerable group of children. | Children (5-10.9 Years old) | Cross sectional | 406 | Anaemia Malnutrition  Vitamin D deficiency |
| 36 | Gupta *et al.* (2007) | Urban slum, namely, ‘Gokul Puri’ in Delhi | To assess the extent of Acute Respiratory Infections (ARI) and Acute Dirrheal Diseases (ADD) in children under 5 by obtaining two week incidence rates; and to study the treatment seeking behaviors of the caretakers for these two illnesses. | Children under 5 years old | Cross sectional | 1307 | Diarrhoea Other respiratory issues |
| 37 | Gupta *et al*. (1998) | At the Treatment-cum-Training Unit (DTTU) of the UCMS and GTB Hospital | To assess the impact of eight different epidemiological factors on frequency of diarrheal episodes amongst children of under-privileged section of society living in the eastern part of the capital city of India. | Mothers of children with acute diarrhea attending the clinic | Cross sectional | 450 | Diarrhoea |
| 38 | Islam *et al*. (2006) | Indore | To examine the health needs and care-seeking behavior of poor slum residents in Indore, India. | Mixed (all age groups) | Mixed design | - | Anaemia Diarrhoea Epilepsy Other respiratory issues |
| 39 | Jain and Aras (2007) | Urban slums of E ward of Mumbai | To assess the epidemiological factors associated with geriatric population and depression. | Elderly | Cross sectional | 196 | Depression |
| 40 | Jain and Mohan (2014) | Meerut City | To explore the problems of adolescents pertaining to sexuality, physical health, tobacco and alcohol use in slums of urban Meerut, and create evidence base for informed planning and decision making by the local health authorities. | Adolescents (Mixed) | Cross sectional | 226 | Sexually transmitted infections |
| 41 | Kanungo *et al.* (2012) | Kolkata | To investigate the differences in clinical, epidemiological and spatial characteristics of the two *Vibrio* species in the urban slums of Kolkata. | Mixed (all age groups) | Case-control | 54, 519 | Cholera Diarrhoea |
| 42 | Karande *et al.* (2002) | Mumbai | To report experience in managing children with acute leptospirosis in a hospital. | Children | Cross sectional | 93 | Leptospirosis |
| 43 | Kattula *et al.* (2015) | Vellore, Tamil Nadu | To identify controllable environmental drivers of intestinal infections amidst a highly contaminated drinking water supply in urban slums and villages of Vellore, Tamil Nadu in southern India. | Mixed (all age groups) | Cohort | 1579 | Diarrhoea |
| 44 | Khopkar *et al.* (2015) | Two slums in Nashik, Maharashtra | To determine the association between blood pressure, mental health and anthropometric status of adolescents from urban slums in Nashik, India. | Adolescents (Mixed) | Cross sectional | 545 | Depression Hypertension Malnutrition Obesity |
| 45 | Lodha *et al.* (2000) | Tertiary hospital in North India | To report a reappearance of cases of microbiologically confirmed diphtheria in a tertiary care hospital in north India. | Children | Case series | 4 | Diphtheria |
| 46 | Madhiwalla (2007) | Not specified | To discuss the healthcare in urban slums in India. | Children | Review article | - | Low birth weight Malnutrition |
| 47 | Manna *et al.* (2013) | Kolkata | Tamil Nadu in southern India. | Children | Cross sectional | 1, 140 | Diarrhoea |
| 48 | Misra *et al.* (2001) | Urban slum colony of Gautam Nagar, South Delhi | To study the lifestyle, anthropometric and metabolic attributes of such a population of low socio-economic strata, we attempted a cross-sectional prevalence survey of obesity, diabetes mellitus, hyperlipidaemia and related lifestyle factors in an urban slum in New Delhi (Delhi Urban Slum Survey), the largest metropolitan city in northern India. | Adults | Cross sectional | 532 | Diabetes  Hypertension Hyperlipidaemia Obesity |
| 49 | Mohan *et al.* (2008) | Six different geographical locations (East, South, North and West/ Central India) | To report the risk factors for self-reported diabetes in Indians from the first national NCD surveillance project conducted in India from April 2003 to March 2005. | Adults | Cross sectional | 44,537 | Diabetes Obesity |
| 50 | Mulgaonkar (1996) | Mumbai | To address the nature and prevalence of gynecological and related morbidity's in the urban slums of Mumbai, India. | Women | Review article | - | Menstrual problems  Reproductive tract infections |
| 51 | Nair *et al.* (2012) | Kolkata | To report the chance finding of *V.cholerae/mimicus* during examination of fecal microbiota of healthy children in an urban slum during a randomized, controlled, probiotic trial. | Children | Case series | 133 | Cholera |
| 52 | Panigrahi and Das (2014) | Bhubaneswar City, Odisha | To ascertain the levels of different forms of undernutrition such as wasting, stunting, and underweight and determine its associates among slum children of 3–9 years residing in Bhubaneswar city, Odisha. | Children | Cross sectional | 249 | Malnutrition |
| 53 | Paul *et al.* (2011) | Kolkata (West Bengal) | To find out the frequency and the type of morbidity among low birth weight (LBW) and Normal birth weight (NBW) infants in an urban slum of Kolkata (West Bengal, India). | Children | Cohort | 126 | Dermatological issues Opthalmalogical issues Other gastrointestinal issues Other respiratory issues |
| 54 | Prashant and Shaw (2009) | Anagal, a slum of Nalgonda town (Andhra Pradesh) | To study the nutritional status of adolescent girls in an urban slum community. | Adolescents (girls) | Cross sectional | 223 | Malnutrition |
| 55 | Puwar *et al.* (2009) | Ahmedabad City | To know the morbidity pattern and health seeking behaviour in a slum area of city. | Mixed (all age groups) | Cross sectional | 685 | Coronary heart disease Rheumatic heart disease Chicken pox Hepatitis Hypertension Malaria, Measles Mental illness Other respiratory issues Typhoid Tuberculosis |
| 56 | Ratho *et al.* (2005) | Peri-urban areas of Chandigarh | To summarise the results of the virological and entomological investigations conducted and meterological parameters collected from the Department of Meterology, Chandigarh. | Mixed (all age groups) | Cross sectional | 218 | Dengue |
| 57 | Sarkar *et al.* (2013) | Ramnaickapalayam, Chinnallapuram, Kaspa and Vasanthapuram - four geographically adjacent, semi-urban slums (Vellore, Tamil Nadu) | To ascertain whether or not the morbidity patterns differed in children drinking bottled water, in addition to capturing the long-term trends in childhood diseases in this population | Children | Quasi-experimental | 160 | Dermatological issues Malnutrition Other gastrointestinal issues Other respiratory issues |
| 58 | Sharma *et al.* (1998) | Two large urban slums (Kathputly Colony and Kusumpur Pahari), Delhi | To determine if there is any relationship between indoor pollution and Acute lower respirotry infection (ALRI) in children, and to identify the risk factors responsible for increases in the severity of ALRI. | Children (newborn) | Cohort | 633 | Malnutrition Pneumonia Other respiratory issues |
| 59 | Shobha *et al*. (2013) | Central Gujarat | To identify the prevalence and type of pathogenic intestinal parasites and to assess the availability of sanitary facilities in the study population to determine the need for control measures. | Mixed (all age groups) | Cross sectional | 1872 | Diarrhoea Parasitic infections |
| 60 | Srivastava *et al.* (2012) | Selected slums of Bareilly City, Uttar-Pradesh | To assess the prevalence of underweight, stunting, and wasting in children of 5 to 15 years old; and to analyze factors associated with malnutrition in children. | Children | Cross sectional | 523 | Anaemia Dental issues ENT issues Malnutrition |
| 61 | Srivastava *et al.* (2008) | A 12-bed Urban Reproductive and Child Health (RCH) center, Lucknow | To assess the difference, if any, in the distribution of neonatal morbidity and care seeking among slum and nonslum dwellers; and to assess variables associated with care-seeking behavior. | Children (newborn) | Cohort | 150 | Congenital anomalies and malformations Dermatological issues Diarrhoea ENT issues Jaundice Ophthalmological issues Other respiratory issues Sepsis |
| 62 | Subbaraman *et al.* (2014) | Non-notified slum, Mumbai | To investigate mental health in Kaula Bandar (KB), a non-notified slum with a population of about 12,000 people situated on a wharf on Mumbai's eastern waterfront. | Adults | Mixed design | 521 | Common mental illness (eg. Depression, anxiety) |
| 63 | Sur *et al.* (2005) | Narkeldanga, Kolkata | To conduct a prospective, community based study in an impoverished urban site in Kolkata in order to measure the burden of cholera, describe its epidemiology, and search for potential risk factors that could be addressed by public health strategies. | Mixed (all age groups) | Prospective study | 62, 329 | Diarrhoea Cholera |
| 64 | Sur *et al.* (2006) | Kolkata (Wards 29 and 30, Narkeldanga) | To estimate the burden of malaria and typhoid fever and to identify risk factors for these diseases. | Mixed (all age groups) | Prospective study | 60, 452 | Fever Malaria Typhoid |
| 65 | Swaminathan and Mukherji (2012) | Delhi, Meerut, Kolkata, Mumbai, Indore, Nagpur, Hyderabad and Chennai | To examine the association between slum residence and nutritional status in women in India by using competing classifications of slum type. | Women | Retrospective study | 15, 000 | Malnutrition Obesity |
| 66 | Vaid *et al.* (2007) | Kaspa Urban Health Centre, Vellore | To know the patterns of morbidity and mortality in children in the local setting in order to formulate appropriate local policies for intervention. | Children (newborn) | Retrospective study | - | Anaemia Congenital anomalies and malformations Diarrhoea Other gastrointestinal issues Fever Hepatitis Hypocalcemia Jaundice Low birth weight Meningitis Obstetric issues Sepsis Pleural effusion Pneumonia Other respiratory issues Renal issues Seizures |
| 67 | Verma *et al.* (2003) | Mumbai | To explore the sexual health problems and treatment seeking behaviour of men in a Mumbai slum population. | Men | Qualitative study | 1344 | Sexually transmitted infections |
| 68 | Wasir *et al.* (2007) | New Delhi | To determine the prevalence of elevated CRP and its associations with obesity and insulin resistance in urban Asian Indian postmenopausal women belonging to a low socio-economic strata. | Women | Cross sectional | 163 | Diabetes Obesity |
